# Questions and Controversies in Neonatal Seizures

**DOI:** 10.3390/children11010040

**Published:** 2023-12-29

**Authors:** Alberto M. Cappellari, Sarah Palumbo, Stefania Margiotta

**Affiliations:** 1 Department of Neuroscience and Mental Health, Foundation IRCCS Ca’ Granda Ospedale Maggiore Policlinico, via Francesco Sforza 35, 20122 Milano, Italy; 2 Postgraduate School of Paediatrics, Department of Pediatrics, University of Milan, 20122 Milano, Italy; sarah.palumbo@unimi.it (S.P.); stefania.margiotta@unimi.it (S.M.)

**Keywords:** neonatal seizures, neonatal epilepsy, neonatal electroencephalography, acute symptomatic seizures

## Abstract

Neonatal seizures are relatively common, but their diagnosis and management remain challenging. We reviewed the scientific literature on neonatal seizures from July 1973 to November 2023. Several parameters were considered, including pathophysiology, diagnostic criteria, electroencephalographic findings and treatment. Recent classification system of seizures and epilepsies in the newborn, as well as treatment recommendations of neonatal seizures, have been proposed. Nonetheless, the approach to neonatal seizures varies among clinicians and centres, including detection, investigation, treatment and follow-up of patients. There are still many issues on the diagnosis and treatment of neonatal seizures, including the meaning or relevance of some electroencephalographic findings, the precise estimation of the seizure burden, the limited efficacy and side effects risk of antiseizure medications, and the best measures to establish the outcome.

## 1. Introduction

Neonatal seizures occur from birth up to 44 weeks of postmenstrual age [1]. While relatively common, the diagnosis and management of neonatal seizures remain challenging [2]. In contrast to seizures in infancy and childhood, those occurring in the neonatal period are often provoked seizures with an acute cause and may be electrographic-only [1]. Therefore, neonatal seizures may not fit easily into seizures and epilepsies classification systems primarily created for children and adults [1]. The International League Against Epilepsy (ILAE) proposed a classification system of seizures and epilepsies in the neonate [1], which facilitates the use of common terminology and assists clinicians in making treatment decisions [3]. Evidence-based recommendations about antiseizure medication (ASM) management in neonates have been also recently developed by ILAE [4]. Nonetheless, the approach to patients with neonatal seizures varies among clinicians and centres, including detection, investigation, treatment and follow-up of these patients [5].

## 2. Materials and Methods

A review of studies on neonatal seizures was performed by literature search on PubMed from July 1973 to November 2023. The goal of this review is not to be all-inclusive. A selection of the more valuable articles on seizures in newborns has been made. We cannot claim to have reported all contributions, but the majority of those included contain substantial references that are worth consulting for a more thorough analysis. We searched for “Neonatal seizures”, “Neonatal epilepsy”, “Neonatal electroencephalography”, and “Acute symptomatic seizures” as our keywords.

## 3. Results

There are many issues on the diagnosis, pathogenesis and treatment of neonatal seizures.

### 3.1. Is the Electrographic Definition of Neonatal Seizures Adequate?

The current definition of neonatal seizure is based on electrographic criteria. An electrographic seizure can be simultaneously coupled with clinical signs (electro-clinical seizure), or not associated with any evident clinical signs (electrographic-only seizure) [1]. A neonatal seizure is defined as “an electrographic event with a pattern characterized by sudden, repetitive, evolving stereotyped waveforms with a beginning and end” [1]. However, the EEG definition of seizures is not appliable to seizure types not associated with repetitive evolving waveforms, such as myoclonic seizures and epileptic spasms. Myoclonic seizures may occur in association with generalized sharp transients, and spasms may be associated with generalized voltage attenuation or generalized sharp transient [6].

The EEG definition relies on monitoring using conventional video-EEG (cEEG). Owing to the challenge due to difficult interpretation of neonatal c-EEG, many neonatal intensive care units (NICU) use amplitude integrated EEG (aEEG), which is a simpler EEG methodology based on a restricted number of channels (1–2 channels) and compressed EEG trace [7,8]. Nonetheless, some studies included newborns with aEEG confirmed seizures for starting treatment [9,10]. Although the ILAE Task Force on Neonatal Seizures confirmed that sensitivity and specificity of aEEG are quite variable, and as such it is not advisable to use it as the primary method for detecting seizures, the authors included in their special report only studies with EEG-confirmed seizures, rarely indicating whether cEEG or aEEG was used [4]. Indeed, due to the limited spatial information and compressed EEG display of aEEG, important information on the specific EEG waveforms is lost [7,8]. In particular, compared with cEEG 20–30% of electrographic seizures will be missed by aEEG [5].

Clinical-only seizures, which were included in the previous definition of neonatal seizures, have been excluded from the current definition, because the majority of these events has been shown to have not an epileptic origin [1,11]. The exclusion of clinical-only seizures raises the issue of possible loss of focal seizures which originate from subcortical cerebral areas like peri-limbic and limbic systems, since these seizures may lack an EEG correlate. At the moment, this notion is neither refutable nor demonstrable [1].

### 3.2. Which Is the Borderland of Brief Rhythmic Discharges?

The old definition of neonatal seizures included a minimal duration of 10 s, which has been removed from the current definition [1]. Brief Rhythmic Discharges (BRDs) are defined as very brief (<10 s) discharges of focal or generalized spiky rhythmic activity, with or without evolution, that are not consistent with normal or benign patterns [12]. Owing to their nature as ictal-interictal continuum, several authors used the term BRDs instead of the term BIRDs (Brief Ictal Rhythmic Discharges), in which the ambiguity of the acronym “I” can either convey ictal or interictal, reflecting a conceptual fuzziness [13]. BRDs may be an indicator of epileptic neuronal networks in the newborn. Furthermore, they can have the same characteristics and they bear the same risk for mortality and neurologic disability as electrographic seizures [14].

The issue of ictal-interictal continuum, which refers to any rhythmic or periodic EEG pattern concerning an ongoing seizure but not meeting formal electrographic criteria to be definitely characterized as ictal, does not concern only neonatal EEG. For example, Periodic Lateralized Epileptiform Discharges (PLEDs) are EEG waves or complexes occurring in sequence at an approximately regular rate [15,16], which may be seen in many diseases [17,18,19]. Significant attention has been devoted to understanding the neurophysiological substrates that underly this EEG pattern and the clinical implication for treatment and outcomes [20]. However, whether PLEDs are an ictal or interictal phenomenon remains controversial [15].

### 3.3. How Seizure Burden Should Be Estimated?

Seizure burden is defined as ictal electrographic activity in a given period of EEG recording [1]. Seizure burden can be measured in minutes per hour and is an expression of the short-term intensity of seizures [21]. A high-seizure burden may be associated with impaired neurodevelopment [10], suggesting that there is an urgent need to control prolonged or recurrent seizures [22]. Considering that BRDs may overlap with electrographic seizures, one should ask if BRDs should also be included when evaluating the seizure burden as an indication to start treatment. This could be an important issue, since the prevalence of BRDs in neonatal studies ranges from 17 to 20% [12,14,23]. Therefore, taking BRDs into account when calculating the seizure burden may significantly modify its value, with subsequent implications in starting medication. Moreover, it is not clear which is the amount of seizure burden requiring treatment. Although a burden of 30 s/h of EEG-proven seizure is required for randomization and treatment in a drug trial according to expert consensus opinion, in clinical practice some physicians administer medication with a seizure burden ≥ 2 min/h, while others treat a single 10 s seizure [24].

Furthermore, although cEEG and aEEG are considered reliable methods for clinical management [4], aEEG has a lower sensitivity in seizure detection [5], possibly leading to underestimation of the total electrographic seizure burden.

### 3.4. Should We Equate the Definition of Neonatal Status Epilepticus to That of Subsequent Ages?

Status epilepticus (SE) is a condition resulting from the failure of mechanisms responsible for seizure cessation or from the initiation of mechanisms, which causes prolonged seizures (after time point t_1_) that can have long-term consequences (after time point t_2_), such as neuronal injury or death and abnormal neuronal networks, based on the type and duration of seizures [25]. Time point t_1_ is the time at which treatment should begin, corresponding to 5 min for tonic-clonic SE, 10 min for focal SE with impaired consciousness, and 10–15 min for absence SE. Time point t_2_ is the time beyond which there is a risk of neuronal damage and alteration of neuronal networks, corresponding to 30 min for tonic-clonic SE, more than 60 min for focal SE with impaired consciousness, and unknown duration for absence SE [25].

Currently, no consensus has been reached about neonatal status epilepticus (NSE) definition [26]. Different definitions of NSE have been suggested and they have all in common a continuous seizure activity lasting for at least 30 min, while they differ in the duration of recurrent seizures, including intermittent seizures lasting for ≥30 min or ≥50% of the recording time [27,28,29].

However, adopted temporal criteria of NSE are highly arbitrary, especially considering that the risk of worse neurological outcome increases above a seizure burden threshold of 12–13 min/h, which is considerably lower than the conventional definition of NSE of 30 min/h [30]. Furthermore, the evidence that EEG-confirmed neonatal seizures essentially arise focally [31] stresses the differences between the time at which long-term consequences may be expected for SE in newborns compared to subsequent ages [25].

### 3.5. When Antiseizure Medications Should Be Started?

New-onset neonatal seizures should be treated as a medical emergency [32]. Current published guidelines for the management of all neonatal seizures recommend starting treatment with anti-seizure-medications (ASM) as soon as possible after seizure recognition, although there are no recommendations on specific timing for treatment [33]. The practice of aEEG/EEG monitoring may be beneficial for babies with high risk for neonatal seizures, including those with hypoxic-ischemic events, cardiac malformations, severe infections and metabolic diseases, as well as very preterm infants. aEEG/EEG monitoring is associated with earlier recognition of seizures, improved detection of subclinical seizures and more precise administration of antiepileptic treatment [34,35].

Despite the improvement in strategies for monitoring, the time-point for starting treatment varies among different studies. Rennie et al. reported that only 11% of electrographic seizures in their study were treated in the first hour following the onset [2]. In the study of Apers et al., 32.1% of patients were treated within 1 h of onset of aEEG confirmed seizure and 19.8% within 1–2 h of onset, whereas most newborns (48.1%) were treated at >2 h after onset [9]. A retrospective study of a cohort of 472 neonates demonstrated that newborns with electrographic seizures treated with ASM in the first hour of seizure onset had the lowest seizure burden and fewer seizures over the following 24 h compared with those treated after 1 h of seizure onset [36]. By contrast, some electrographic seizures registered with aEEG/EEG monitoring in extremely preterm infants (in which the prevalence of seizures may be very high), in particular when brief and subclinical, such as those associated with intraventricular haemorrhages and early morbidity, show no clear association with long-term outcome [37,38], suggesting that ASM may not be necessary for these subclinical seizures.

Therefore, the issue of time point for starting treatment in neonatal seizures is still to be better defined. Large prospective studies are necessary, owing to the variability in how neonatal seizures are diagnosed and managed, and a consensus is needed [39,40,41,42].

### 3.6. Which Is the Optimal First-Line ASM?

Newborns need to be carefully considered when designing a treatment plan, as both seizures and their treatment may affect the developing brain [32]. While there are several published guidelines and there is an international consensus on the management of acute seizures and status epilepticus in children and adults, with proven effective drugs [43,44,45], evidence-based guidelines for neonatal seizures have been limited due to a lack of randomized controlled trials (RCTs) and the difficulty of conducting accurate studies on newborns [24,46]. Only recently have the guidelines and consensus-based recommendations on treatment of neonatal seizures been published by ILAE [4]. Until that moment, the management of neonatal seizures has been largely driven by the experience and preferences of physicians [47,48].

According to 2011 guidelines on neonatal seizures [33,49], phenobarbital (PHB) remains the most commonly used agent for first-line treatment of neonatal seizures [47], with a response rate of approximately 43% [22,50]. However, PHB is associated with significant side effects, including sedation, abnormal neuronal apoptosis in rodent models, and memory problems when used long term [32,51].

A RCT study assessing the efficacy of PHB and phenytoin (PHT) for the treatment of seizures in term and preterm neonates reported no difference in efficacy between the two drugs as first-line treatment [4,50]. A retrospective nonrandomized study on full-term newborns found that both fosphenytoin and PHB were equally efficacious as a first-line ASM in neonatal seizure control. Furthermore, infants treated with fosphenytoin had significantly better neurodevelopmental outcomes, as compared to PHB. However, the limited sample size hindered the ability to identify slight or moderate differences in drugs that could have clinical significance [52].

In the last years, growing interest has arisen in the use of levetiracetam (LEV) as a first-line treatment for neonatal seizures because it comes in oral and intravenous forms, it is reasonably safe for older children and infants, and pharmacokinetic data in newborns are available [32,53,54]. There are only a few studies assessing the efficacy of LEV versus PHB for the treatment of neonatal seizures, and some of them are retrospective [32,55] or use clinical seizures as the outcome measure [56]. A recent meta-analysis of fourteen studies assessing 1188 neonates showed that LEV might not be more effective than PHB (low certainty of evidence), although it is associated with a lower risk of adverse events (moderate certainty of evidence) [57]. Recently, the NEOLEV2 trial (NCT01720667), which is the first randomized controlled trial (RCT) comparing the efficacy and adverse effects of LEV to PHB as first line treatment in neonatal seizures, established that LEV is not as effective as PHB for initial neonatal seizure management [58]. PHB as a first-line treatment is probably more effective than LEV in controlling seizures after the first loading dose and maximal loading dose of ASM [59]. Although more adverse effects have been reported in subjects randomly assigned to PHB compared to LEV, they were not statistically significant [58].

Recent ILAE guidelines and consensus-based recommendations on treatment of seizures in the neonate reported that PHB should be the first-line ASM, while sodium channel blockers, such as PHT or carbamazepine (CBZ), may be the first-line ASM if channelopathy is the likely cause for seizures due to family history [4]. At the moment, LEV is not indicated as a first-line drug for acute symptomatic neonatal seizures [32] and long-term follow-up studies in newborns treated with LEV are essential to evaluate its impact on long-term seizures and cognitive outcome [60].

### 3.7. Which Is the Rationale for Using Phenobarbital in Neonatal Seizures?

In the early postnatal period, endogenously released γ-aminobutyric acid (GABA) is excitatory and contributes to the decreased ictal epileptiform activity threshold [61]. In the neonatal rodent brain, GABAergic drugs can have excitatory effects, potentially aggravating seizures and their long-term consequences. This possible deleterious effect in brain areas associated with little behavioral expression at neonatal age raises important questions, owing to a possible delayed behavioral and clinical expression of the damage [62]. Both PHB and midazolam treatment of SE in postnatal day 7 rat pups have been associated with increased acute neuronal injury in several brain regions, raising questions about the safety of their use in clinical practice [62]. On the other hand, PHB and midazolam partially suppressed the seizures in rat model of birth asphyxia [63]. Adding further complexity to the issue, PHB reduces initial ictal-like events, but increases them when applied after the formation of a mirror focus caused by the propagation of recurrent ictal-like events [64].

Although physiological studies of the GABA system in rodent neonates provide a potential conceptual framework for the possible relationship between GABAergic drugs and brain injury, their clinical significance in clinical practice is uncertain [62]. However, this possibility should not be overlooked, also considering that the response rate to PHB was only approximately 43% [22,50]. It is worth noting that blinded studies of PHB in childhood epilepsy did not show significant differences in behavioral or cognitive adverse effects compared to other AEDs, which is in contrast to the excess of such adverse effects reported in studies open to observer bias [65].

### 3.8. Which Is the Optimal Second-Line ASM?

A descriptive analysis of 11 neonatal seizure management pathways at 11 level IV neonatal intensive care units (NICUs) in the United States showed that recommended second-line ASM was split between fosphenytoin (5/11) and LEV (2/11), while the other four pathways suggested making a decision based on the clinical context [47].

Fosphenytoin is currently being used more often than PHT because of its fewer side effects related to extravasation and cardiotoxicity [66]. However, also fosphenytoin can lead to hypotension or cardiac arrhythmias, and the pharmacokinetics of PHT can be unpredictable in neonates, making the drug less widely used than PHB as a first-line agent [32,47]. LEV is an attractive alternative as it has been associated with few adverse effects in neonates [55,67,68]. Despite lack of studies supporting its use at that time, a 2007 survey reported that LEV was recommended by 47% of pediatric neurologists as off-label drug for the treatment of neonatal seizures [69]. The current division in pathway recommendations between fosphenytoin and LEV as second-line neonatal ASMs raises the need of future studies directly comparing their effectiveness and safety [47].

Infusion of benzodiazepines, such as midazolam, or lidocaine are commonly used as second- or third-line drugs, whether seizures are refractory to repeated loading doses of standard ASMs [22,32]. Anyway, the efficacy of benzodiazepines has not been thoroughly studied for neonatal seizures, despite their widely accepted use as first-line treatment for seizures in older children and adults [66]. Midazolam infusion is a treatment option for refractory seizures caused by acute brain injury or status epilepticus [32], but it may be associated with hypotension in nearly 10% of newborn and other adverse events such as transient hypoxia, apnea and tachycardia [70,71]. Moreover, in very preterm newborns, exposure to midazolam has found to be inversely associated with hippocampal volume and cognitive outcomes [72]. Lidocaine infusions may be indicated for refractory acute symptomatic seizures. However, there are concerns about cardiac toxicity, so that lidocaine is contraindicated in neonates with congenital heart disease or those who have been already treated with PHT [73].

Recent ILAE consensus-based recommendations reported that PHT or LEV may be used as a second-line in neonates with seizures not responding to first-line ASM, with a preference for LEV in newborns with cardiac disorders. Other possible options include midazolam or lidocaine, while sodium channel blockers (PHT or CBZ) may be used if channelopathy is suspected because of clinical or EEG features. However, the authors recognized that the choice of second-line ASM therapy remains unclear [4]. Nonetheless, some useful flow-charts with common lines of treatment have been published [32,66,74,75].

### 3.9. When Antiseizure Medications Should Be Stopped?

Long-term treatment with ASM is usually indicated for neonatal epilepsies [32,76]. Only recently have the consensus-based recommendations on the duration of treatment with ASM for acute symptomatic seizures been published by ILAE [4]. Until that moment there was no clear consensus on the ideal duration of treatment following cessation of acute symptomatic seizures [22,32]. The World Health Organization (WHO) recommends that ASM discontinuation should be considered in neonates with acute symptomatic seizures in the presence of normal neurological exam and/or normal EEG after at least 72 h of seizure freedom [33]. However, there remains significant variability in how long ASM are continued in neonates with acute symptomatic seizures [49,77]. The risk of postneonatal epilepsy in the first years of life is less than 25% for neonates with acute symptomatic seizures, and postneonatal epilepsies do not necessarily respond to the medications prescribed in the neonatal period [77,78,79,80]. Furthermore, a growing body of research indicates that earlier stopping ASM for acute symptomatic seizures in newborns does not usually result in a recurrence of seizures, a higher risk of childhood epilepsy, or a worsening of developmental outcomes [76,81,82]. Shellhaas et al. performed a prospective, observational cohort study of consecutive newborns with seizures treated at the 7 sites of the Neonatal Seizure Registry. In this study, although some sites discontinued ASM, in most or all neonates with acute symptomatic seizures, the majority of neonates with acute seizures (73%) were discharged home on ASM, including those with a low seizure burden or even those without confirmed electrographic seizures on cEEG (continuous video-electroencephalogram) recordings. These results emphasize the urgent need for a rigorous study of the optimal duration of treatment for newborns with acute symptomatic seizures [77]. Indeed, Keene et al. showed that there was consensus in the pathway recommendations at NICUs to strongly consider ASM discontinuation before hospital discharge, although exact timing remains variable [47]. This variability likely reflects the challenge of balancing support of seizure cessation with concern for both acute side effects and long- term adverse effects of using ASM [47,83,84,85].

Recent ILAE consensus-based recommendations reported that, following cessation of acute provoked seizures (electroclinical or electrographic) without evidence for neonatal-onset epilepsy, ASMs should be discontinued before discharge home, regardless of MRI or EEG findings [4].

### 3.10. Is Seizure-Induced Damage Worse Than ASM-Induced Damage?

Immature brain is susceptible to the damage provoked by seizures [86]. There is evidence about negative neurodevelopmental outcomes associated with neonatal seizures, which include epilepsy, developmental delay, psychomotor deficits, and cerebral palsy [87,88,89], but it is not clear whether seizures per se can influence long-term outcomes or are an indicator of the severity of underlying conditions [87,90,91]. Furthermore, most animal studies demonstrated that seizures can impair brain growth during the perinatal period when they are severe or repeated [86,92,93,94,95,96], while most neonatal seizures have a relatively brief duration of 1–3 min [24,97]. This topic could assume even greater relevance in preterm infants, who are known to experience seizures of shorter duration compared to term infants [97,98], although the former have more seizures [99].

The use of some ASM can be complicated by both systemic side effects and neurotoxicity. In particular, PHB, which is considered the first line treatment of neonatal seizures [33], has been associated with hypotension and respiratory depression [100], as well as brain development and cognitive impairment [101]. Studies on animal models demonstrated that PHB generates substantial increase in developmental apoptosis, produces serious damage in striatal function with subsequent durable deficits in reversal learning behavior, and causes abiding behavioral changes [102]. The vulnerability period to the proapoptotic effect of ASM coincides with the brain growth period, which in the rat extends to the first 2 weeks of postnatal life. In humans, the similar period begins in the third trimester of gestation and extends to several years after birth [84].

Further knowledge of seizure-induced damage versus ASM-induced damage is necessary when considering treatment of neonatal seizures, and a balance between these factors should be pondered.

### 3.11. Which Should Be the Outcomes for Future Studies?

Most studies have used seizure cessation as the primary outcome measure [58,66]. However, the kind of seizure response to assess ASM efficacy has varied in different studies, since some studies target complete seizure cessation while others report percent seizure reduction or response within a specific time, such as one or two hours after acute seizures onset. This variability limits the possibility of comparing the results across different studies [49]. Furthermore, while most studies have concentrated on the acute suppression of seizures, the most effective choice for ASM should be based on long-term developmental outcome [58,66]. A drug that is less effective in stopping seizures but leads to a better developmental outcome due to a neuroprotective effect or lack of neurotoxicity may be the first-choice option for treatment [56,58]. Nevertheless, long-term developmental outcome measures are commonly not assessed, despite the critical importance of this information to families and clinicians [103,104,105]. On the other hand, neonatal seizures have a negative impact on developmental outcome [31], while no significant association between ASM treatment duration and functional development at age 24 months has been reported [76].

## 4. Discussion

There is great progress in the diagnosis and management of neonatal seizures, including the development of an automated seizure detection system [66,106,107], as well as the availability of precision medicine for some genetic etiologies [66,108,109]. Automated seizure detection system can be especially useful in warning bedside personnel about the likelihood of seizures, even though it is currently not feasible to fully replace the expert human reviewer [66]. However, several issues should be considered in future research and clinical trials. A thorough assessment of the seizure burden both before and after treatment is required to avoid the mistaken belief that seizures are improved from ASM rather than they are spontaneously resolved [110]. However, even though aggressive seizure therapy can reduce the overall seizure burden, it is still unclear if this reduction will ultimately lead to a better neurodevelopmental outcome [66,111]. Although the majority of clinical trials focus on the acute suppression of seizures, the best ASM selection should be based on long-term developmental outcome, taking into account the risk of neurotoxicities from the ASM themselves [62,84,100,101,102,112]. The issue of outcome measures for infants who have been treated with ASM does not concern only neonatal seizures. It is perhaps worth noting that in the EPISTOP trial on infants with Tuberous Sclerosis Complex, preventive treatment with vigabatrin reduced the risk and severity of epilepsy, while the improvement in intellectual disability in the preventive versus conventional treatment group was not significant at age 2 years [113]. Therefore, it is essential to understand optimal ASM selection for neonatal seizures, as well as treatment duration after cessation of seizures [66]. Indeed, ILAE reports on classification system of seizures and epilepsies in the neonate [1] and treatment recommendations of neonatal seizures [4] represent a giant step to assist clinicians in making treatment decisions.

## 5. Conclusions

There are still many questions and controversies on the diagnosis and treatment of neonatal seizures, including the meaning or relevance of some EEG features, the accurate quantification of the seizure burden, the balance between efficacy and side effects of antiseizure medications, and the measures to establish the outcome. Until now, the diagnosis and management of neonatal seizures remain challenging, and the approach to them varies among clinicians and centres.

## Data Availability

Not applicable.
